# Maternal High Folic Acid Supplement Promotes Glucose Intolerance and Insulin Resistance in Male Mouse Offspring Fed a High-Fat Diet

**DOI:** 10.3390/ijms15046298

**Published:** 2014-04-14

**Authors:** Yifan Huang, Yonghan He, Xiaowei Sun, Yujie He, Ying Li, Changhao Sun

**Affiliations:** 1 Department of Nutrition and Food Hygiene, Public Health College, Harbin Medical University, Harbin 150086, China; E-Mails: yyzhyf@hotmail.com (Y.Hu.); sunxw919@163.com (X.S.); savannah870912@gmail.com (Y.He); liying__helen@126.com (Y.L.); 2 State Key Laboratory of Genetic Resources and Evolution, Kunming Institute of Zoology, the Chinese Academy of Sciences, Kunming 650223, China; E-Mail: heyonghan@mail.kiz.ac.cn

**Keywords:** folate, epigenetics, metabolic syndrome, gestational nutrition supplement

## Abstract

Maternal nutrition may influence metabolic profiles in offspring. We aimed to investigate the effect of maternal folic acid supplement on glucose metabolism in mouse offspring fed a high-fat diet (HFD). Sixty C57BL/6 female mice were randomly assigned into three dietary groups and fed the AIN-93G diet containing 2 (control), 5 (recommended folic acid supplement, RFolS) or 40 (high folic acid supplement, HFolS) mg folic acid/kg of diet. All male offspring were fed HFD for eight weeks. Physiological, biochemical and genetic variables were measured. Before HFD feeding, developmental variables and metabolic profiles were comparable among each offspring group. However, after eight weeks of HFD feeding, the offspring of HFolS dams (Off-HFolS) were more vulnerable to suffer from obesity (*p* = 0.009), glucose intolerance (*p* < 0.001) and insulin resistance (*p* < 0.001), compared with the controls. Off-HFolS had reduced serum adiponectin concentration, accompanied with decreased adiponectin mRNA level but increased global DNA methylation level in white adipose tissue. In conclusion, our results suggest maternal HFolS exacerbates the detrimental effect of HFD on glucose intolerance and insulin resistance in male offspring, implying that HFolS during pregnancy should be adopted cautiously in the general population of pregnant women to avoid potential deleterious effect on the metabolic diseases in their offspring.

## 1. Introduction

High prevalence of type 2 diabetes, which is developed from glucose disturbance and insulin resistance, has become a global health problem [1]. As part of a classical western lifestyle, the high-fat diet (HFD) has been proven to be closely associated with glucose disturbance and insulin resistance. Habitual HFD generally induces dyslipidaemia, glucose disturbance, abnormal secretion of inflammatory factors, and further promotes insulin resistance and diabetes [2]. In addition to HFD, as suggested by accumulating evidence, unbalanced maternal nutrition also contributes to the incidence and development of glucose disturbance and insulin resistance in offspring [3]. Intrauterine exposure to improper nutrition affects the expression of foetal genes, and programs the future risk of metabolic diseases in their later life [4], involving epigenetic changes such as aberrant DNA methylation as probable mechanisms.

Folate is a water-soluble vitamin that plays a fundamental role in the epigenetic pathway as a methyl donor [5]. Because maternal folate supplement lowers risk of birth defects and Alzheimer’s disease, many countries encourage periconceptional folic acid (synthetic form of folate) supplement [6]. Meanwhile, mandatory folic acid fortification has been implemented by more than fifty countries [7]. Nevertheless, considering that folic acid is more bioactive than folate *in vivo* [8], and increasing evidence supports that maternal folic acid supplement (FolS) influences normal DNA methylation in offspring [9,10], potential adverse effect of folic acid on the foetus should be taken into account. Several studies revealed that high maternal folic acid consumption increased the risk of allergy disease [11] and mammary tumours [12], and we found two studies on possible detrimental effect of maternal FolS on glucose metabolism in offspring particularly noteworthy: one is that in mouse models, gestational methyl donor supplement significantly increased body fat in offspring [13], although this has not yet been proven in the general population; moreover, a recent population-based study observed that low maternal vitamin B12 accompanied with high folate concentration during pregnancy was associated with higher insulin resistance in offspring [14]. In addition, some studies showed that gestational multivitamin supplement affected the deleterious effect of postweaning HFD on glucose regulation, obesity and metabolic syndrome in offspring, involving altered epigenetic regulation of related genes as possible mechanisms [15–17]. Folate plays a central role in normal epigenetic processes [5], but whether maternal FolS could also similarly alter the deleterious effect of post-adulthood HFD on glucose metabolism and insulin resistance in offspring is still poorly understood.

Therefore, we designed this study to test the hypothesis that maternal FolS may alter the effect of HFD on glucose metabolism and insulin resistance in rodent offspring.

## 2. Results

### 2.1. Maternal Status and Folate Concentrations

**Figure 1.**
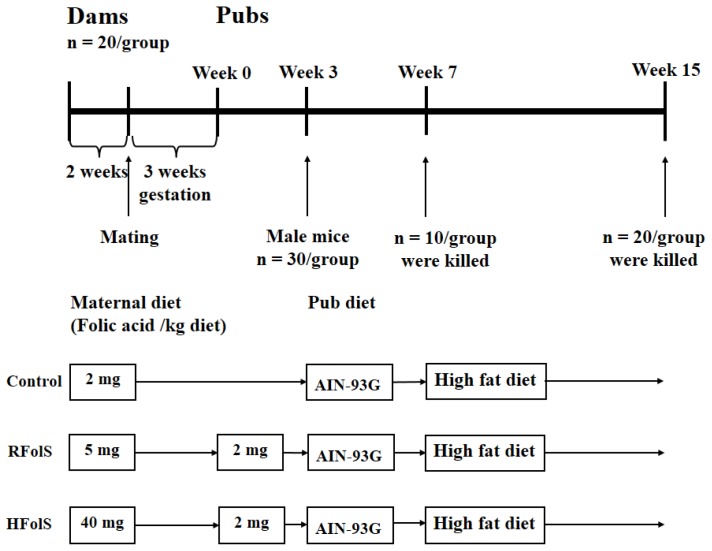
The whole design of our study. At weaning (week 3), after 30/group male mice were randomly selected, the rest of the male, and all female mice were killed. Abbreviations: HFolS, high folic acid supplement; RFolS, recommended folic acid supplement.

Before and during pregnancy, no differences in body weight gain and dietary intake were observed between each maternal group, while both RFolS and HFolS groups had significantly higher daily folic acid intakes than the control group (Table S1). After two weeks of pregestational FolS (Figure 1), both RFolS (8.51 ± 0.72 *versus* 4.37 ± 0.68 mmol/L, *p* < 0.001) and HFolS (18.23 ± 2.13 *versus* 4.37 ± 0.68 mmol/L, *p* < 0.001) dams had higher serum folate concentrations compared with the controls. Serum homocysteine concentrations were lower in HFolS (13.5 ± 1.3 *versus* 16.7 ± 1.5 nmol/mL, *p* < 0.001) and RFolS (14.8 ± 1.2 *versus* 16.7 ± 1.5 nmol/mL, *p* = 0.010) groups, compared with the control group.

### 2.2. Body Weight, Body Composition and Dietary Intake

**Figure 2.**
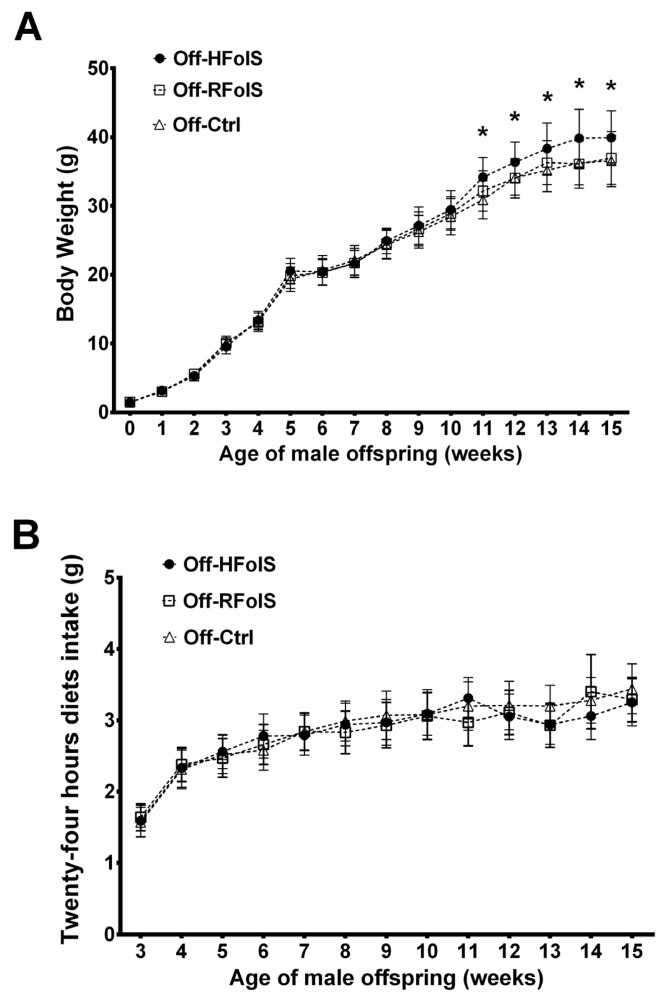
Postnatal body weight (**A**) and postweaning 24 h food intake (**B**) of the male offspring in each group. * *p* < 0.05, compared with the Off-Ctrl group. Abbreviations: Off-Ctrl, offspring of the control dams; Off-HFolS, offspring of the high folic acid supplement dams; Off-RFolS, offspring of the recommended folic acid supplement dams.

Maternal FolS affected neither litter size nor birth weight. At baseline, body weight and body composition were comparable among offspring groups, while after eight weeks of HFD feeding (Figure 1), the Off-HFolS but not the Off-RFolS group had higher body weight and marginally higher adiposity index compared with the Off-Ctrl group (Figure 2A and Table 1). Dietary intakes were comparable among the Off-Ctrl, Off-RFolS and Off-HFolS groups from 3 weeks to 15 weeks (Figure 2B).

### 2.3. Glucose Response, Insulin Resistance and Lipid Variables

**Figure 3.**
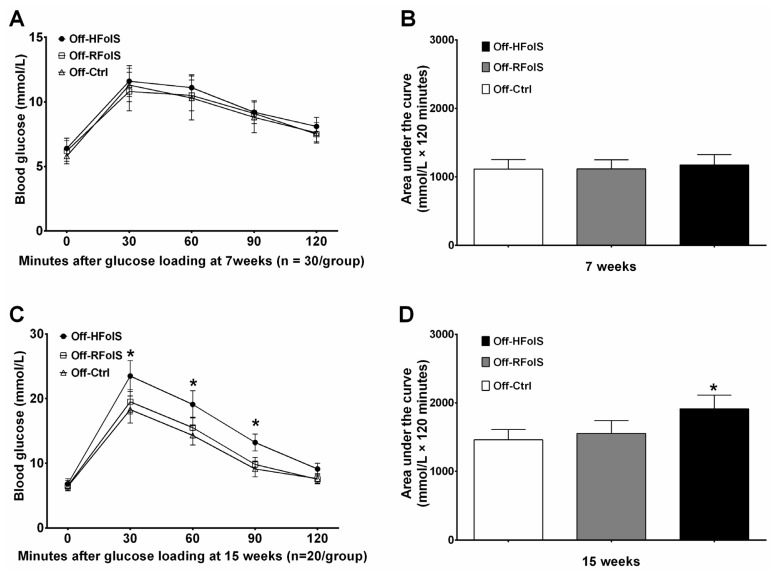
The results of glucose tolerance test and the related area under the curve in the offspring at seven weeks (**A** and **B**) and 15 weeks (**C** and **D**). * *p* < 0.05, compared with the Off-Ctrl. Abbreviations: Off-Ctrl, offspring of the control dams; Off-HFolS, offspring of the high folic acid supplement dams; Off-RFolS, offspring of the recommended folic acid supplement dams.

Maternal FolS did not affect fasting glucose, insulin resistance and lipid variables in the offspring of seven weeks old. However, after eight weeks of HFD feeding, the Off-HFolS group had significantly higher post loading blood glucose (area under the curve: 1913 ± 201 *versus* 1461 ± 152 mmol/L × 120 min, *p* < 0.001) and insulin resistance (HOMA-IR: 7.39 ± 1.41 *versus* 2.16 ± 0.43, *p* < 0.001), compared with the Off-Ctrl group (Table 1 and Figure 3).

### 2.4. Inflammatory Factors and Adipokines

**Figure 4.**
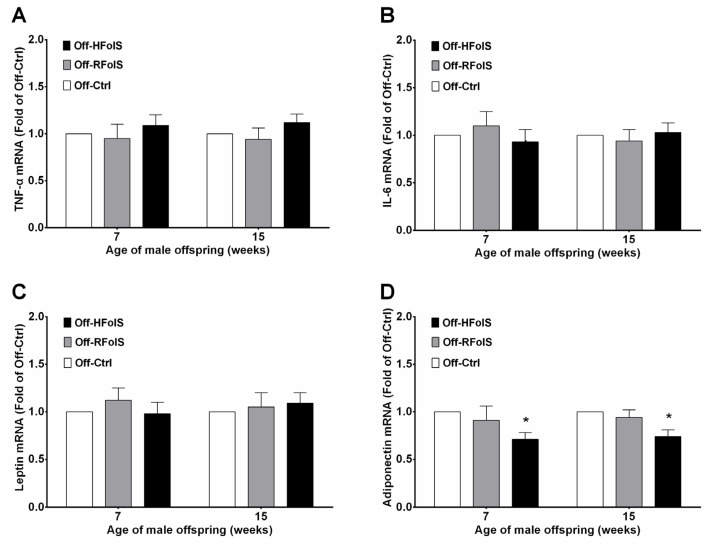
The relative mRNA levels of *TNF-α* (**A**); *IL-6* (**B**); *leptin* (**C**) and *adiponectin* (**D**) in white adipose tissue at seven weeks (*n* = 10/group) and 15 weeks (*n* = 20/group). * *p* < 0.05, compared with the Off-Ctrl. Abbreviations: Off-Ctrl, offspring of the control dams; Off-HFolS, offspring of the high folic acid supplement dams; Off-RFolS, offspring of the recommended folic acid supplement dams.

We failed to find significant changes in TNF-α, IL-6 and leptin in the offspring between groups before and after HFD feeding, although serum TNF-α level was marginally higher in the Off-HFolS group than in the Off-Ctrl group (at seven weeks, 19.84 ± 1.73 *versus* 18.42 ± 1.44 pg/mL, *p* = 0.063; at 15 weeks, 24.58 ± 2.41 *versus* 22.82 ± 3.12 pg/mL, *p* = 0.091). However, the Off-HFolS mice had a consistently decreased trend of serum adiponectin, regardless of HFD (at seven weeks, 7.72 ± 1.12 *versus* 8.93 ± 0.97 mg/L, *p* = 0.019; at 15 weeks, 5.21 ± 0.52 *versus* 6.68 ± 0.76 mg/L, *p* < 0.001) (Table 1). We further investigated the relative mRNA expression levels of *TNF-α*, *IL-6*, *leptin* and *adiponectin* in white adipose tissue, and found that maternal HFolS significantly decreased the expression of *adiponectin* by 29% in the offspring at seven weeks, and by 26% at 15 weeks. No significant changes in *TNF-α*, *IL-6* and *leptin* expressions were observed among offspring groups (Figure 4).

### 2.5. Global Methylation

Both maternal RFolS and HFolS increased the global methylation levels of white adipose tissue in the offspring, while only HFolS did significantly (at seven weeks, 7.8% ± 0.7% *versus* 5.7% ± 1.1%, *p* < 0.001; at 15 weeks, 8.1% ± 1.4% *versus* 5.6% ± 0.9%, *p* < 0.001).

## 3. Discussion

In this study, we mainly found that maternal HFolS, but not RFolS, exacerbated the deleterious effect of HFD on glucose tolerance and insulin resistance in male mouse offspring, indicating that adverse effect of maternal FolS depend on the dose of supplemental folic acid.

### 3.1. FolS, Growth and Development

Although substantial evidence support that maternal low folic acid consumption and folate deficiency status affect the growth and development of offspring [18], whether maternal FolS shows a similar effect is still unclear. Birth weight is an important pregnancy outcome linked to the health in later life. A previous systematic review based on nine population studies found that gestational folate supplementation doses from 0.25 to 5 mg per day significantly increased birth weight compared with the control group [6]. Despite similar findings from epidemiological studies, investigations on animal models have shown conflicting results [8,19,20]. Our results showed that both Off-HFolS and Off-RFolS did not differ in birth weight or litter size compared with the Off-Ctrl, being consistent with a previous study on rats [19]. However, it should be noted that there was higher body weight gain in the Off-HFolS group after HFD feeding. As an essential component of metabolic syndrome, obesity is prevalent among many countries, which is positively associated with hypertension, insulin resistance and diabetes [21]. Different obese phenotypes among three offspring groups in our study indicate probable detrimental effect of maternal high folic acid dose consumption on the development of obesity, especially for those exposed to HFD in adulthood. The exact mechanisms for this detrimental effect were still unclear, but it may not be fully explained by the epigenetic changes in the pro-opiomelanocortin promoter caused by folate [22], since postweaning dietary intake was comparable among groups at all times.

### 3.2. FolS, Insulin Resistance, Inflammatory Cytokines and Adipokines

The detrimental effect of maternal HFolS on insulin resistance in our animal model was somewhat in agreement with a recent epidemiological study in India, in which the authors found that maternal folate concentration, rather than vitamin B12, was positively associated with insulin resistance in their children [23]. However, the mechanisms were still unclear. Since elevated inflammatory factors were closely associated with impaired glucose tolerance and insulin resistance [24], we assessed the expression of *TNF-α* and *IL-6* to investigate whether changes in inflammatory status could link the maternal HFolS to insulin resistance. The marginally but consistently increased trend of serum TNF-α in the Off-HFolS was intriguing, but should be interpreted cautiously, because the changes in corresponding mRNA levels in white adipose tissue were not significant. At present we cannot rule out the possibility that it is increased body weight and adiposity index caused by maternal HFolS, rather than maternal HFolS itself, that contributes to the elevated TNF-α level in the offspring. Besides inflammatory factors, we determined adipokines because they also affect the pathological process of insulin resistance [25]. Adipokines or adipocytokines, such as leptin and adiponectin, are hormones mainly produced by adipocytes. The release of adipokines regulates glucose metabolism, and influences pathological processes of insulin resistance and type 2 diabetes [26,27]. Leptin and adiponectin were studied because of the evidence that leptin affected insulin resistance in mice independent of changing body weight [28], whereas adiponectin promoted the survival and function of islet β cells [29] and improved peripheral insulin sensitivity [30]. Interestingly, serum adiponectin level was significantly lower in the Off-HFolS mice than in the Off-Ctrl ones, even if before HFD feeding. What is more, the consistently decreased adiponectin mRNA level in white adipose tissue was also observed in the Off-HFolS group. These results suggest that the altered adiponectin expression was not likely to be caused by HFD, rather intrauterine exposure to HFolS. Some epidemiological studies showed that blood adiponectin level was inversely associated with insulin resistance [31,32], and adiponectin-knockout mice were easy to exhibit insulin resistance [33]. Taken together, it is reasonable to suppose that maternal HFolS affects the expression of adiponectin in offspring, and further exacerbates the deleterious effect of HFD on glucose tolerance and insulin resistance.

### 3.3. FolS and DNA Methylation

DNA methylation of cytosine in the cytosine-guanine dinucleotide sequences is heritable, and aberrant DNA methylation usually leads to abnormal expression of related genes [34]. Although determination of global DNA methylation is less convincing than specific DNA methylation, we still determined it because accumulating evidence over the past decades revealed that maternal FolS led to not only hypermethylation but also hypomethylation in offspring [35]. Increased global methylation of white adipose tissue in the Off-HFolS group indirectly provides a promising pathway to link the maternal FolS and repression of adiponectin; it also indirectly explains that why maternal RFolS did not similarly affect the expression of adiponectin in offspring as the HFolS did: Dose-dependent effect probably exists in the association between maternal folate concentrations and offspring methylation, hence 5 mg folic acid/kg of diet supplement may not be enough to significantly change the specific methylation of adiponectin in the foetus.

### 3.4. Clinical Relevance

Our results are relevant to the use of folic acid supplement in the general population of pregnant women. The dose of 2.5-fold folic acid in this study is equivalent to an adult intake of 1000 μg/day according to the new recommended daily allowance [36]; while the dose of 20-fold folic acid is equivalent to an adult intake of 4 or 8 mg/day according to the earlier and new requirement [36,37]. The data derived from RFolS group indicate that at least for glucose tolerance and insulin resistance, no significant evidence was found to support adverse effect of maternal FolS of 1000 μg/day in human offspring. The dose of 8 mg/day is above the usual folate recommendation for the prevention of neural tube defect recurrence. Since extra folate can be eliminated in the urine, folic acid is generally considered safe for pregnant women themselves [38]. However, the results derived from the HFolS group in this study suggest that high folic acid consumption in pregnancy probably increases the risk of insulin resistance and glucose disturbance in offspring, especially for those accustomed to long-term HFD in adulthood. In addition, the altered global DNA methylation caused by maternal HFolS is also concerning, since results from animal and epidemiological studies suggest that aberrant DNA methylation is related to various diseases in later life [34].

### 3.5. Limitations and Strengths

There are some limitations that should be taken into account in this study. First and foremost, due to the species difference, our results should not be directly extrapolated to human; it is still necessary to further evaluate the relationship between maternal FolS, HFD in adulthood and insulin resistance in the general population; secondly, we did not provide the data on old offspring, and the mechanistic information is not enough; thirdly, not all inflammatory factors and adipokines were included in our study, hence we cannot exclude the possibility that other unexamined factors affected by HFolS are involved in the association between maternal FolS and insulin resistance of offspring. Despite these limitations, the results still indicate that HFolS during pregnancy should be adopted cautiously in general pregnant women to avoid potential deleterious effect of metabolic diseases in their offspring.

## 4. Experimental Section

### 4.1. Selection of Folic Acid Dose

At first, we designed three maternal folic acid diet groups based on a normal AIN-93G diet [39] containing 2 (control), 5 (recommended folic acid supplement, RFolS) or 40 (high folic acid supplement, HFolS) mg folic acid/kg of diet. The lowest level of folic acid (2 mg/kg) in the control group is generally accepted as the basic requirement for rodents and was selected to represent the requirement (400 μg/day) for nonpregnant women. The medium level (5 mg/kg) of folic acid in the RFolS group, which is 2.5 times the requirement for rodents, represents approximate total folate intake (800–1000 μg/day) from mandatory folic acid fortification in North American populations [40], and the tolerable upper intake level (1000 μg/day) for pregnant women [36]. The highest level (40 mg/kg) of folic acid in the HFolS group is 20 times the requirement for a normal mouse. This is because to prevent neural tube defect recurrence, the highest folate recommendation is 4 mg/day for high-risk pregnant women, which is about 20 times the earlier requirement (180–200 μg/day) for nonpregnant women [37].

### 4.2. Animals and Design

Animal care and experimental procedures in this study were approved by the Animal Experimental Committee of Harbin Medical University. Virgin C57BL/6 mice (30 males, 60 females, seven weeks old) were purchased from Charles River Inc. (Vital River Ltd., Beijing, China). After arrival, all mice were housed in our animal facility under clean conditions with a standardized environment (21.0 ± 1.5 °C, 50% ± 10% relative humidity, and 12:12 h light-dark cycles). All mice were housed individually and allowed recovery for seven days with *ad libitum* access to food and water.

Dams were randomly divided into control, RFolS or HFolS groups (*n* = 20 per group) and fed the above-described diet before and throughout pregnancy. Male mice used for breeding were fed the control AIN-93G diet before mating, and the same diet with female mice during mating. Male mice were removed once the gestation was successful, according to the observation of vaginal plug. At birth, intragroup each litter was culled to five pups to minimize the difference in milk availability, and the rest of the pups were discarded. Briefly, litters with more than five pups were culled to five pups randomly, excess pups were collected together and randomly distributed to the litters with less than five pups. We compared intragroup mean birth weight of pups before and after this process to avoid possible selection bias. During lactation, pups remained with the dams, and the dams were only fed control AIN-93G diet. At weaning, all male pups in each group were given random numbers by sorting body weight. The male pups with the lowest 30 random numbers were selected from each group and fed control AIN-93G diet individually until seven weeks old, and the others were killed. To rule out the potential influence of female hormones such as estrogen on results [41], all female pups were discarded. Detailed randomized procedures at 0 and 3 weeks were presented in Figure S1. At seven weeks of age, 10 male pups per group were randomly selected and killed, whereas the rest of the 20 male per group were fed HFD until the 15th week. Therefore, three offspring groups existed in our final study: Offspring of the control dams (Off-Ctrl), *n* = 20; offspring of the recommended folic acid supplement dams (Off-RFolS), *n* = 20; offspring of the high folic acid supplement dams (Off-HFolS), *n* = 20. All pups were killed at the end of 15 weeks of age. The whole study design was portrayed in Figure 1, and the differences between the control AIN-93G diet and HFD were presented in Table S2.

### 4.3. Observational Parameters and Glucose Tolerance Test

Body weight was measured weekly at the same time in each day (8:30–9:30 a.m.). Twenty-four hour food intake of the offspring was carefully measured twice every week from 3 to 15 weeks of age, and determined as the average value. The glucose tolerance test was performed at seven weeks and 15 weeks. After fasting for five hours with water available *ad libitum*, an intraperitoneal injection of sterile glucose (0.5 g glucose/mL, 2 mg/g of body weight) was administered. Blood recovered from the tip of the tail at five different time points (0, 30, 60, 90 and 120 min after injection) was tested with a Lifescan One Touch Ultra glucometer (Johnson & Johnson, Northridge, CA, USA). The area under the curve was calculated by the trapezoid rule (GraphPad Prism software 5.01, San Diego, CA, USA).

### 4.4. Sampling and Biomedical Measurements

Blood was collected from the eyeball after five hours fast and centrifuged at 3000 rpm for 10 min at 4 °C to obtain serum. Serum and samples were obtained from pups at 3, 7 and 15 weeks of age, and immediately stored at −80 °C until further test. Adiposity index was calculated as adiposity index = (intra − abdominal fat pad + subcutaneous fat pad) × 100%/animal body weight. The serum folic acid concentrations were measured by AMEKO enzyme-linked immunosorbent assay (ELISA) kits (Shanghai Lianshuo Biological Technology Co., Ltd., Shanghai, China), and serum homocysteine concentrations were measured by ELISA kits (Cusabio, Wuhan, China). Serum fasting glucose, total cholesterol, high and low density lipoprotein cholesterol concentrations were measured with standard enzymatic methods in an auto-analyser (AUTOLAB PM 4000, AMS Corporation, Rome, Italy). Serum insulin level was measured in an auto-analyser using commercial kits (Centaur, Bayer Corporation, Bayer Leverkusen, Germany). Homeostasis model assessment of insulin (HOMA-IR) was calculated as HOMA-IR = Fasting glucose (mmol/L) × Fasting insulin (mIU/L)/22.5. Levels of tumour necrosis factor-α (TNF-α) and interleukin-6 (IL-6) were assessed using ELISA kits (Beijing 4A Biotech Co., Ltd., Beijing, China). Levels of adiponectin and leptin were assessed using ELISA assay kits (Uscn Life Science, Wuhan, China).

### 4.5. Quantitative Real-Time PCR

Total mRNA of white adipose tissues was isolated from samples using TRIzol Reagent (Invitrogen, Life Technologies Corp., Carlsbad, CA, USA) and cDNA was synthesized using a cDNA Reverse Transcription Kit (Applied Biosystems Inc., Foster City, CA, USA) according to the manufacturer’s instructions. The purity and quantity of the isolated RNA were spectrophotometrically determined using the GeneQuant 1300 spectrophotometer (GE Healthcare Bio-sciences AB, Uppsala, Sweden). The mRNA levels were determined using the Applied Biosystems 7500 Fast Real-Time PCR System with SYBR Green PCR Master mix (Applied Biosystems Inc., Foster City, CA, USA). The quantitative real-time PCR amplification procedure was briefly described as follows: samples were predenatured at 95 °C for 10 min followed by 40 cycles of amplification consisting of 15 s at 95 °C, 30 s at 55 °C, and 30 s at 72 °C; β-actin was used as the internal control. To verify the accuracy and specificity, all reactions were performed at least in triplicate, followed by melting curve analysis. The relative quantification of mRNA was evaluated using the 2^−ΔΔ^*^C^*^t^ method [42]. Dilution curves were adopted to assess the PCR efficiency as proposed by Bustin *et al*. [43]. The sequences of primers (Sangon Biotech Co., Ltd., Shanghai, China) were given in Table S3.

### 4.6. Global Methylation

Genomic DNA of white adipose tissues was isolated using QIAamp DNA Mini Kits (Qiagen, Valencia, CA, USA) and determined using the GeneQuant 1300 spectrophotometer (GE Healthcare Bio-sciences AB, Uppsala, Sweden). Global DNA methylation was quantified using MethylFlash Methylated DNA Quantification Kits (Epigentek, Farmingdale, NY, USA) according to the manufacturer’s instructions. Briefly, DNA was bound to strip wells that were specifically treated to have a high DNA affinity. The methylated fraction of DNA was detected using capture and detection antibodies and then quantified colorimetrically by reading the absorbance at 450 nm in a microplate reader (Molecular Devices, MD-SpectraMax M5, Sunnyvale, CA, USA). The amount and percentage of methylated DNA were calculated by measured optical density values. All investigations were performed in triplicate using two independent replicates.

### 4.7. Statistical Analysis

Data are presented as mean ± standard deviation. The differences in continuous variables among groups were compared by analysis of variance (ANOVA) followed by the *post hoc* Bonferroni method, and each mouse was treated as individual for statistical analysis. Statistical analyses were performed with SPSS software version 20.0 (IBM Co., Armonk, NY, USA). All *p* values less than 0.05 were considered statistically significant, and *p* values between 0.05 and 0.10 were considered to marginally statistically significant.

## 5. Conclusions

Overall, our findings suggest that maternal HFolS significantly exacerbates the deleterious effect of HFD on glucose tolerance and insulin resistance in male mouse offspring, implying that HFolS during pregnancy should be adopted cautiously in the general population of pregnant women to avoid potential deleterious effect of metabolic diseases in their offspring.

## Supplementary Information



## Figures and Tables

**Table 1.** Body composition and biomedical variables in offspring before and after HFD feeding.

Variable	Off-Ctrl	Off-RFolS	Off-HFolS	*p*
Litter size	6.2 ± 0.9	5.8 ± 1.2	6.1 ± 1.0	0.454

7 weeks				

Numbers	10	10	10	
Intra-abdominal fat pad, g	0.31 ± 0.03	0.34 ± 0.03	0.32 ± 0.04	0.147
Subcutaneous fat pad, g	0.13 ± 0.02	0.13 ± 0.01	0.14 ± 0.02	0.344
Adiposity index, %	1.99 ± 0.21	2.17 ± 0.20	2.13 ± 0.19	0.127
Fasting glucose, mmol/L	5.94 ± 0.83	6.15 ± 0.61	6.39 ± 0.81	0.424
Insulin, mIU/L	7.31 ± 1.53	7.57 ± 1.13	7.67 ± 1.27	0.822
HOMA-IR	1.93 ± 0.50	2.08 ± 0.42	2.20 ± 0.54	0.475
Total cholesterol, mmol/L	2.67 ± 0.52	2.59 ± 0.77	3.09 ± 0.85	0.273
HDLC, mmol/L	1.73 ± 0.21	1.67 ± 0.21	1.60 ± 0.14	0.323
LDLC, mmol/L	0.60 ± 0.10	0.61 ± 0.05	0.63 ± 0.10	0.735
TNF-α, pg/mL	18.42 ± 1.44	18.55 ± 0.97	19.84 ± 1.73	0.063
IL-6, pg/mL	15.41 ± 1.12	16.13 ± 0.86	15.92 ± 1.04	0.279
Leptin, pg/mL	461.73 ± 38.15	487.47 ± 69.33	512.82 ± 78.71	0.226
Adiponectin, mg/L	8.93 ± 0.97	8.37 ± 0.84	7.72 ± 1.12 *	0.035

15 weeks				

Numbers	20	20	20	
Intra-abdominal fat pad, g	0.78 ± 0.08	0.82 ± 0.07	0.87 ± 0.08 *	0.002
Subcutaneous fat pad, g	0.47 ± 0.05	0.44 ± 0.03	0.59 ± 0.06 *	<0.001
Adiposity index, %	3.41 ± 0.33	3.45 ± 0.38	3.68 ± 0.41	0.057
Fasting glucose, mmol/L	6.33 ± 0.87	6.47 ± 0.66	6.82 ± 0.68	0.109
Insulin, mIU/L	7.66 ± 1.21	7.71 ± 1.30	24.49 ± 4.47 *	<0.001
HOMA-IR	2.16 ± 0.43	2.22 ± 0.42	7.39 ± 1.41 *	<0.001
Total cholesterol, mmol/L	3.98 ± 0.41	3.97 ± 0.30	4.12 ± 0.43	0.392
HDLC, mmol/L	1.61 ± 0.18	1.62 ± 0.22	1.63 ± 0.19	0.950
LDLC, mmol/L	0.58 ± 0.10	0.58 ± 0.07	0.61 ± 0.08	0.435
TNF-α, pg/mL	22.82 ± 3.12	22.91 ± 2.84	24.58 ± 2.41	0.091
IL-6, pg/mL	20.35 ± 1.84	21.31 ± 1.47	21.54 ± 2.01	0.092
Leptin, pg/mL	867.40 ± 102.62	843.92 ± 88.75	916.26 ± 92.38	0.056
Adiponectin, mg/L	6.68 ± 0.76	6.20 ± 0.73	5.21 ± 0.52 *	<0.001

*p* values are derived from analysis of variance with the *post hoc* Bonferroni method, and

* indicates *p <* 0.05, compared with Off-Ctrl. Adiposity index was calculated as adiposity index = (intra-abdominal fat pad + subcutaneous fat pad) × 100%/animal body weight. HOMA-IR = fasting glucose × insulin/22.5. Abbreviations: HDLC, high density lipoprotein cholesterol; HFD, high-fat diet; HOMA-IR, homeostasis model of assessment for insulin resistance index; IL-6, interleukin-6; LDLC, low density lipoprotein cholesterol; mIU, milli-international unit; Off-Ctrl, offspring of the control dams; Off-HFolS, offspring of the high folic acid supplement dams; Off-RFolS, offspring of the recommended folic acid supplement dams; TNF-α, tumour necrosis factor-α.

## Abbreviations

ANOVA: analysis of variance
FolS: folic acid supplement
HFD: high-fat diet
HFolS: high folic acid supplement
HOMA-IR: homeostasis model assessment of insulin resistance
IL-6: interleukin-6
Off-Ctrl: offspring of the control dams
Off-HFolS: offspring of the high folic acid supplement dams
Off-RFolS: offspring of the recommended folic acid supplement dams
RFolS: recommended folic acid supplement
TNF-α: tumour necrosis factor-α
