# Supplementary material for: Maternal High Folic Acid Supplement Promotes Glucose Intolerance and Insulin Resistance in Male Mouse Offspring Fed a High-Fat Diet
Source: Int J Mol Sci. 2014 Apr 14;15(4):6298–313. doi: 10.3390/ijms15046298 (PMC4013629; doi:10.3390/ijms15046298)
Supplement: Supplementary file 1 [file ijms-15-06298-s001.pdf]

Supplementary Information

Table S1. The maternal folic acid intakes before and during pregnancy among groups (μg/day).

	**Ctrl**	**RFolS**	**HFolS**
**Number**	**20**	**20**	**20**
**Week**			
−2	5.78 ± 0.42 $^{\#}$	14.79 ± 1.26 *	117.74 ± 10.57 $^{*,\#}$
−1	5.92 ± 0.54 $^{\#}$	15.01 ± 1.27 *	121.43 ± 9.05 $^{*,\#}$
1	5.96 ± 0.46 $^{\#}$	15.19 ± 1.41 *	119.53 ± 11.22 $^{*,\#}$
2	6.48 ± 0.46 $^{\#}$	16.13 ± 1.19 *	129.15 ± 11.98 $^{*,\#}$
3	10.91 ± 1.03 $^{\#}$	26.68 ± 2.05 *	208.77 ± 16.74 $^{*,\#}$

Daily folic acid intake (μg/day) = Folic acid contents in diets (μg/g) × daily food intake (g/day). Data are compared by analysis of variance with the *post hoc* Bonferroni method. * $p < 0.05$, compared with Ctrl; $^{\#}$ $p < 0.05$, compared with RFolS. Negative sign represents the weeks before pregnancy. Abbreviations: Ctrl, Control; HFolS, High folic acid supplement; RFolS, recommended folic acid supplement.

Table S2. Formulated control and obesogenic diet.

**Ingredient**	**Control AIN-93G diet (g)**	**High fat diet (g)**
Cornstarch	397.5	287.5
Casein	200.0	200.0
Dextrinized cornstarch	132.0	132.0
Sucrose	100.0	100.0
Soybean oil	70.0	30.0
Lard [a]	0.0	150.0
Fiber	50.0	50.0
Mineral mix (AIN-93G-MX)	35.0	35.0
Vitamin mix (AIN-93-VX)	10.0	10.0
L-Cystine	3.0	3.0
Choline bitartrate	2.5	0.0
Cholate	0.0	2.5

[a] The actual fatty acid composition of lard is 1.4% 14:0, 24.8% 16:0, 2.2% 16:1, 13.2% 18:0, 44.3% 18:1, 13.8% 18:2 and 0.1% 18:3.

Table S3. Primer pairs used in present study.

**Gene**	**Primer**
Adiponectin	Forward: GTCAGTGGATCTGACGACACCAA<br>Reverse: ATGCCTGCCATCCAACCTG
IL-6	Forward: TCCAGTTGCCTTCTTGGGAC<br>Reverse: GTGTAATTAAGCCTCCGACTTG
Leptin	Forward: GATGACACCAAAACCCTCATC<br>Reverse: GCCACCACCTCTGTGGAGTAG
TNF-α	Forward: CAGGAGGGAGAACAGAAACTCCA<br>Reverse: CCTGGTTGGCTGCTTGCTT
Actin	Forward: CATCCGTAAAGACCTCTATGCCAAC<br>Reverse: ATGGAGCCACCGATCCACA

Figure S1. The detailed procedures of random selection at delivery and weaning.

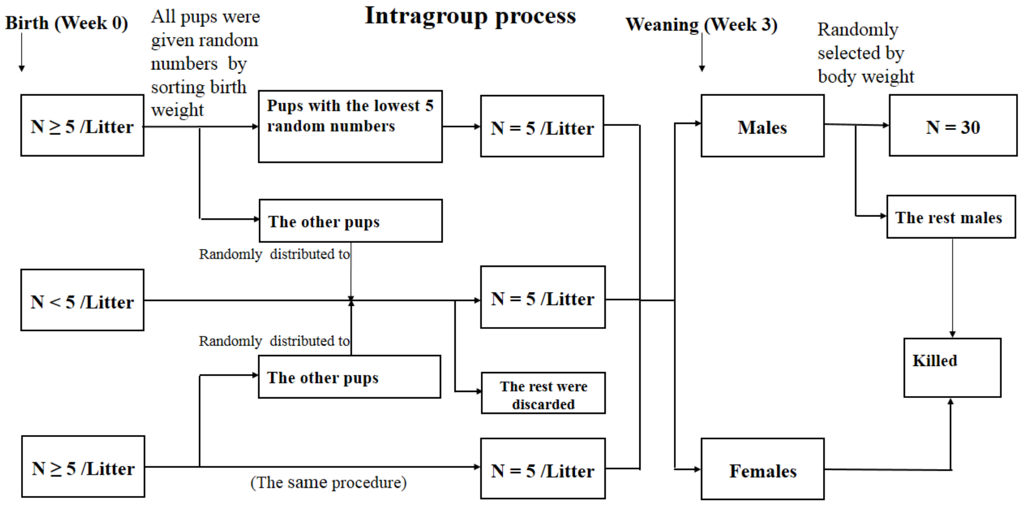

© 2014 by the authors; licensee MDPI, Basel, Switzerland. This article is an open access article distributed under the terms and conditions of the Creative Commons Attribution license (http://creativecommons.org/licenses/by/3.0/).
